# Real-world retrospective study of immune checkpoint inhibitors in combination with radiotherapy or chemoradiotherapy as a bladder-sparing treatment strategy for muscle-invasive bladder urothelial cancer

**DOI:** 10.3389/fimmu.2023.1162580

**Published:** 2023-05-22

**Authors:** Chao Xu, Wen Zou, Lei Zhang, Ran Xu, Yuan Li, Yeqian Feng, Runtian Zhao, Yinhuai Wang, Xianling Liu, Jingjing Wang

**Affiliations:** ^1^ Department of Oncology, The Second Xiangya Hospital, Central South University, Changsha, China; ^2^ Department of Urology, The Second Xiangya Hospital, Central South University, Changsha, China; ^3^ Department of Oncology, Guilin Hospital of the Second Xiangya Hospital, Central South University, Guilin, China

**Keywords:** bladder cancer, muscle-invasive bladder urothelial cancer, bladder-sparing treatment, programmed cell death protein-1 inhibitors, radiotherapy

## Abstract

**Background:**

Recent developments in MIBC treatment suggest good efficacy of bladder sparing treatment combined with immune checkpoint inhibitor. However, there is no standard treatment mode. A retrospective analysis was conducted to reveal the efficacy and safety of PD-1 inhibitor in combination with radiotherapy or chemoradiotherapy.

**Methods:**

We retrospectively analyzed 25 patients with MIBC T2-T3N0M0 disease who were unfit or unwilling to undergo RC. These patients underwent the maximum TURBT followed by PD-1 inhibitor (Tislelizumab or Toripalimab) in combination with radiotherapy or chemoradiotherapy (gemcitabine plus cisplatin) between April 2020 and May 2022. The primary outcome was clinical complete response (cCR) rate. The secondary outcomes were disease free survival (DFS) and overall survival (OS).

**Results:**

Revised: Of 25 patients, 22 were T2 (88%), while 3 were T3 (12%). The median age is 65 years (51–80). Twenty-one patients had programmed cell death ligand 1 (PD-L1) combined positive score (CPS) of 1 or more, and 4 patients had CPS<1 or unknown. Sixteen patients received chemoradiotherapy. Tislelizumab and Toripalimab were administered to 19 and 6 patients, respectively. The median number of cycles of immunotherapy was 8. Twenty-three patients (92%) achieved cCR. Following a median of 13 months of follow-up (range, 5-34 months), 1-year DFS and OS rate were 92% and 96%, respectively. In the univariate analysis, T stage significantly influenced OS and ORR, and efficacy evaluation significantly influenced OS, DFS, and ORR. The expression of PD-L1 and chemotherapy had no effect on prognosis. In the multivariate analysis, no independent prognostic factors were found. Grade 3 or 4 adverse events (AE) were reported in 35.7% patients.

**Conclusions:**

Bladder sparing therapy with PD-1 inhibitor in combination with radiotherapy or chemoradiotherapy is feasible, safe, and highly effective for patients who were unfit or unwilling to undergo RC.

## Introduction

Bladder cancer is the tenth most common cancer globally and the second most common genitourinary malignancy (1). Currently, non-muscle invasive bladder cancer (NMIBC) comprises approximately 70% of bladder cancers, whereas muscle-invasive bladder cancer (MIBC) comprises the remaining 30% of the localized disease (2). MIBC is invasive and has high morbidity and mortality and poor prognosis. Currently, neoadjuvant chemotherapy with radical cystectomy (RC) is the standard treatment option for MIBC, with a 5-year survival rate of approximately 60% (3). However, it has been reported that more than 50% of patients with MIBC do not prefer aggressive surgical treatment (4). This preference may be associated with a significant reduction in the quality of life (QOL) of postoperative patients, especially for older adult patients (5, 6). The bladder-sparing protocol (BSP) expands the treatment options for patients with MIBC who are unwilling to perform or ineligible for RC. Trimodality therapy (TMT), which includes maximum transurethral resection of bladder tumor (TURBT) and concurrent chemoradiotherapy (CCRT), is currently the most studied and widely recognized bladder preservation strategy (7). Moreover, some patients who are weak or have special conditions can also choose TURBT combined with radiotherapy, TURBT combined with chemotherapy, or single therapy, including radical TUR, chemotherapy, and radiotherapy alone; however, these treatments are generally recognized as less effective than TMT in terms of bladder preservation (8). It is of great significance to develop more bladder-preserving strategies to address the needs of different populations and improve prognosis.

Immune checkpoint inhibitors (ICIs) have provided new prospects for the treatment of MIBC. Currently, programmed cell death protein-1 (PD-1), programmed death-ligand 1 (PD-L1), and cytotoxic T-lymphocyte-associated antigen 4 inhibitors are the most studied ICIs, and various PD-1/PD-L1 inhibitors have been approved for the treatment of advanced bladder cancer (9, 10). Because of the limited efficacy of single-agent immunotherapy, research has focused on the combination of chemoradiotherapy and immunotherapy and shown that they have a good synergistic effect. Therefore, combination therapy has become a major trend (11, 12). According to the traditional bladder-preserving strategy, there are three main types of treatment combinations comprising immunotherapy: (i) immunotherapy combined with CRT, (ii) immunotherapy combined with radiotherapy, and (iii) immunotherapy combined with chemotherapy (13–16). Although these treatment modes show great potential, no standard treatment mode has been established. Therefore, we conducted a retrospective real-world study to determine the feasibility, efficacy, and safety of PD-1 inhibitors combined with radiotherapy or chemotherapy.

## Materials and methods

### Patients

A cohort of patients diagnosed with MIBC was retrospectively examined at the Second Xiangya Hospital of Central South University between April 2020 and May 2022. The inclusion criteria were as follows: (1) those with a pathological diagnosis of T2-T3N0M0 MIBC, (2) those who were ineligible or unwilling to undergo RC surgery and selected TURBT followed by the administration of PD-1 inhibitors in addition to radiotherapy with or without chemotherapy, and (3) those with complete follow-up data. The exclusion criteria were as follows: (1) those with tumors other than MIBC, infectious diseases, hematological diseases, or severe liver or renal dysfunction and (2) those who underwent partial cystectomy. The last follow-up was conducted on October 1, 2022. Disease stage was defined according to the American Joint Committee on Cancer (AJCC) tumor-lymph node-metastasis (TNM) Staging System, 8th edition. The study design was approved by the appropriate ethics review board.

### Treatment

Patients first underwent maximum TURBT and received PD-1 inhibitor and radiotherapy with or without chemotherapy 2 to 3 weeks later. PD-1 inhibitors included 200 mg of tirelizumab administered or 240 mg of toripalimab both administered intravenously every 3 weeks for an expected duration of 1 year. Some patients with good tolerance received concurrent chemotherapy with gemcitabine (1000 mg/m2 d1,6) plus cisplatin (50–75 mg/m2) every three weeks for four cycles. Intensity-modulated radiation therapy was used for external irradiation, which was planned with the Varian Eclipse Treatment Planning System version 11.0 (Varian Medical Systems, Palo Alto, CA, USA) and delivered with 6-MV X-rays using Varian 23EX (Varian Medical Systems).

Gross tumor volume (GTV) includes bladder tumors and positive lymph nodes, which, was confirmed using computed tomography (CT), magnetic resonance imaging (MRI), cystoscope, or positron-emission tomography (PET), and the clinical target volume (CTV) included the whole bladder and regional-nodal basin. The planning target volume was delineated by margins of 7–10 mm around the GTV and CTV. Cone-beam CT was performed weekly. The CTV dose was 45–50 Gy with 1.8 or 2 Gy administered daily, and the GTV dose was 66 Gy, with 2 Gy administered daily. Imaging (CT, MRI, cystoscopy, or PET) and cystoscopy biopsies were performed 12 weeks after radiotherapy and every three months during follow-up to evaluate the therapeutic effect.

### Data collection

Clinical information, including age, sex, physical status, T stage, PD-L1 expression, pathological differentiation, treatment strategy, and imaging results, was collected.

### Statistical analysis

SPSS software version 26.0 (SPSS, Inc., Chicago, IL, USA) was used for statistical analysis. The major endpoint was the clinical complete response (cCR) rate 12 weeks after radiotherapy. The secondary endpoints were disease-free survival (DFS), OS, and objective response rate (ORR). cCR was defined as the absence of any tumor during TURBT (12 weeks after radiotherapy) and no evidence of a local tumor, lymph node metastasis, or distant metastasis on CT or MRI. OS was defined as survival from the date of diagnosis to death or final follow-up. DFS was defined as survival from the date of diagnosis to disease recurrence, death (for any reason), or final follow-up. ORR refers to the proportion of patients whose tumors shrank to a certain size and remained at that size for a certain time, including complete remission and partial remission. ORR was evaluated using the evaluation standard of solid tumor efficacy version 1.1 (RECIST v1.1).

A Kaplan–Meier univariate analysis was performed to assess the 1-year OS and DFS rates. Statistical differences between survival curves were evaluated using the log-rank test. Multivariate Cox proportional hazard models were created using factors influencing prognosis, as shown in previous studies (17). Fisher’s exact test and χ2 test were used to determine whether there was a correlation between two variables. P-values <0.05 were considered statistically significant. Hazard ratios (HRs) and 95% confidence intervals (CIs) were estimated to assess the magnitude of risk.

## Results

### Patient and treatment characteristics

A total of 25 patients were included in the analysis. The median follow-up time was 13 (range, 5–34) months. Patient characteristics are summarized in **Table 1**. The median age of included patients was 65 (range, 51–80 years) years. Twenty-two patients had T2 (88%) disease, whereas three had T3 (12%) disease. Twenty-one patients had a PD-L1 combined positive score (CPS) of ≥1. Four patients had a CPS <1 or unknown CPS. Seventeen patients received chemotherapy, and the median number of cycles was 3. Nineteen and six patients were treated with tirelizumab and toripalimab, respectively. The median number of immune cycles administered was 8.

**Table 1:** Clinical characteristics of patients with MIBC.

Variable	Number (%)
**Total**	25 (100)
**Median Age (y)**	65
**Median BMI**	24.61
Sex	
Male	21 (84.0)
Female	4 (16.0)
PS	
0	16 (64)
1	9 (36)
T Stage	
T2	21 (84.0)
T3	4 (16.0)
PD-L1 Expression	
Unknown	1 (4.0)
PD-L1 CPS<1	3 (12.0)
PD-L1 CPS=1-9	11 (44.0)
PD-L1 CPS≥10	10 (40.0)
Histological Grade	
High	17 (68.0)
Low	8 (32.0)
**Median ICI Cycle**	8
ICI	
Tislelizumab	19 (76.0)
Toripalimab	6 (24.0)
**Median Chemotherapy cycles**	3
Chemotherapy	
Gem+Cisplatin	17 (68.0)
None	8 (32.0)

### Prognostic analysis

**Figure 1:**
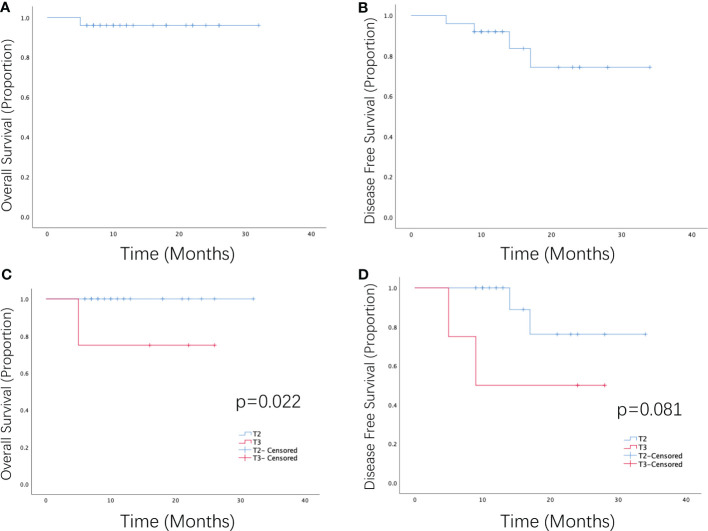
Survival curve of patients with MIBC receiving PD-1 inhibitors combined with radiotherapy or chemotherapy as a bladder-sparing treatment strategy. **(A)** Overall survival and **(B)** disease-free survival of patients with MIBC who received PD-1 inhibitors in combination with radiotherapy or chemoradiotherapy. **(C)** Overall survival and **(D)** disease-free survival of patients with T2–T3 MIBC who received PD-1 inhibitors in combination with radiotherapy or chemoradiotherapy.

Twenty-five patients underwent bladder-sparing treatment combined with ICI administration, 23 patients (92%) had cCR, and 2 (8%) had PR. The ORR rate was 100%. The 1-year OS and DFS rates were 96% and 92%, respectively (**Figures 1A, B**). In the univariate analysis, T stage significantly influenced OS and ORR, and efficacy evaluation significantly influenced OS, DFS, and ORR (**Table 2**). The 1-year OS and DFS rates of patients with T2 disease were both 100%, which were better than those of patients with T3 disease, whose 1-year OS and DFS rates were 75% and 50% (**Figures 1C, D**), respectively. All patients with T2 disease and 50% of patients with T3 disease had cCR. The 1-year OS and DFS rates of patients with CR were both 100%, which were better than those of patients with PR, whose 1-year OS and DFS rates were 50% and 0%, respectively. PD-L1 expression and chemotherapy had no effect on the OS, DFS, or ORR. T stage, PD-L1 expression, ICIs, and chemotherapy, which have been shown in previous studies to affect prognosis, were included in the multivariate analysis, but no independent prognostic factors were found (**Supplementary Table 1**).

**Table 2:** Univariate analysis of overall survival, disease-free survival rates, and objective response rates for all patients.

Variable	Univariate Analysis					Objective Response		
	1-year OS rate (%)	P-value	1-year DFS rate (%)		P-value	CR	PR	P-value
**Total**	96		92			23 (92%)	2 (8%)	
Age, years								
≤65	92.3		92.3			12	1	
>65	100	0.337	91.7		0.903	11	1	0.953
BMI								
≤24.61	92.3		84.6			11	2	
>24.61	100	0.337	100		0.226	12	0	0.157
T Stage								
T2	100		100			21	0	
T3	75.0	0.022	50		0.081	2	2	0.001
Sex								
Male	95.2		90.5			19	2	
Female	100	0.663	100		0.433	4	0	0.520
PD-L1 Expression								
PD-L1 CPS<1 or unknown	100		100			4	0	
PD-L1 CPS≥ 1	95.2	0.663	90.5		0.433	19	2	0.52
Histological Grade								
High	94.1		94.1			16	1	
Low	100	0.493	87.5		0.574	7	1	0.569
Chemotherapy								
Yes	94.1		88.2			15	2	
No	100	0.493	100		0.254	8	0	0.312
ICI								
Tislelizumab	100		94.7			18	5	
Toripalimab	83.3	0.075	83.3		0.737	1	1	0.369
Efficacy Evaluation								
CR	100		100					
PR	50	0.001	0		0.000			

### Outcome and toxicity

Among the 25 patients, 3 patients had intravesical recurrence, 2 of whom received systemic treatment, 1 of whom was received TURBT again. In addition, 1 patient died of multiple metastases.

In total, 92% of patients in this study experienced AEs. Among these patients, 35.7% experienced grade 3 or 4 AEs, and the common adverse events were fatigue (14.3%), cystitis (7.1%), nausea and vomiting (14.3%), thrombocytopenia (14.3%), and leukopenia (7.1%). Immune-related AEs were mainly fatigue (50%), hypothyroidism (44%), and skin pruritus (40%). Except for fatigue, all AEs were of grade 1 or 2 (**Table 3**).

**Table 3:** Adverse event profiles.

Adverse Effect	Patients (%)
**TRAE**	23 (92)
G3-4 TRAE	4 (16)
**IRAE**	16 (64)
G3-4 IRAE	2 (8)
**Hematologic**	7 (28)
G3-4	3 (12)
**Neutropenia**	6 (24)
G3-4	3 (12)
**Thrombocytopenia**	2 (8)
G3-4	0 (0)
**Hypothyroidism**	11 (44)
G3-4	2 (8)
**Radiation proctitis**	12 (48)
G3-4	0 (0)
**Radiation cystitis**	16 (64)
G3-4	1 (4)
**Pruritus and Erythra**	6 (24)
G3-4	0 (0)
**Elevated liver enzymes**	5 (20)
G3-4	0 (0)
**fatigue**	6 (24)
G3-4	0 (0)

## Discussion

In the field of bladder-conserving therapy, many types of bladder-preserving treatment modes are combined with immunotherapy, including TMT, radiotherapy, and chemotherapy. Among these, immunotherapy combined with TMT is the most widely studied. A study on pembrolizumab combined with CCRT for MIBC included 28 patients, and the CR rate was 88% (18). Another study on pembrolizumab combined with gemcitabine and hypofractionated radiotherapy for bladder-preserving therapy of MIBC has demonstrated that the 1-year BIDFS rate was 77% and that the CR rate at 12 weeks was 83%–100% (19). In this study, 17 (68%) patients received TMT combined with PD-1 inhibitors; the 1-year OS and DFS rates were 93.8% and 100%, respectively, and the cCR rate was 88.2%. Thus, our results are consistent with the aforementioned findings and have better short-term effects than traditional bladder-sparing therapy, demonstrating the feasibility and effectiveness of ICIs combined with TMT.

Studies on immunotherapy combined with radiotherapy comprised patients who were unable to tolerate chemotherapy. A study of concurrent nivolumab and radiotherapy for older adult patients with MIBC with a median age of 78 years showed that 6 of 14 patients had CR (20). Another phase II study of durvalumab combined with concurrent RT for patients with a median age of 74 years, lymph node positivity, and an inability to undergo complete cystoscopic tumor resection, showed that the rate of CR was 50% (10 CR, 3 PR, 1 SD, and 6 PD), that of 1-year progression-free survival (PFS) was 73%, and that of 1-year and 2-year OS was 83.8% and 76.8%, respectively (21). A study of atezolizumab combined with radiotherapy for patients with a median age of 78.6 years showed that the pathologic complete remission (pCR) rate was 100% (22). Treatment with durvalumab and tremelimumab combined with radiotherapy for patients with MIBC also had a positive effect. The CR rate was 81% (26/32), and the 6-month bladder intact DFS (BI-DFS), DFS, and OS rates were 76%, 80%, and 93%, respectively (15). These studies showed that immunotherapy combined with radiotherapy for older adult patients with MIBC having poor tolerance is feasible and effective. Before the era of immunotherapy, a study showed that patients with T1–T4 bladder cancer received a BSP of radiotherapy alone or radiotherapy and cisplatin chemotherapy and that 63.5% of them achieved CR (23). Therefore, the data from some studies demonstrate that immunotherapy combined with radiotherapy is not worse than TMT combined with radiotherapy and better than radiotherapy alone or radiochemotherapy in terms of outcomes. In this study, 8 older adult patients did not receive chemotherapy. And there was no difference between patients who received chemotherapy and those who did not. However, the sample size of this study is too small, which may affect the results. Whether the combination of ICI can replace the sensitization effect of chemotherapy still needs further study.

Anyway, compared with traditional bladder-preserving treatment, the preliminary CR rate of immunotherapy combined with TMT was 83–100%, while the CR rate of traditional TMT was approximately 75% (20). The CR rate of immunotherapy combined with radiotherapy was 42.85–100%, and radiotherapy alone was approximately 64% (21). It suggests that patients benefit more from combined immunotherapy bladder-preserving treatment.

The immune drugs involved in the bladder preservation study included pembrolizumab, nivolumab, ipilimumab, atezolizumab, durvalumab, tremelimumab, tislelizumab, and toripalimab. Until now, to our knowledge, no head-to-head studies have compared the differences between these drugs. This study enrolled patients who received tislelizumab as well as those who received toripalimab and found that there was no difference in efficacy and prognosis between these two drugs. Tislelizumab and toripalimab have been approved by the China Food and Drug Administration for the posterior line treatment of bladder urothelial carcinoma (24, 25). One study compared the efficacies of neoadjuvant chemotherapy plus ICIs and chemotherapy alone with that of MIBC bladder preservation therapy. ICIs included pembrolizumab, tislelizumab, and toripalimab. The CR and DCR rates in the combined immunotherapy group and chemotherapy alone groups were 50% and 0%, and 95.5% and 66.7% (P=0.003), respectively (26). Another study on tislelizumab combined with nab-paclitaxel for MIBC bladder preservation showed that the pCR rate and 1-year relapse-free survival rate were 77.3% and 82%, respectively (27). These studies demonstrated that these two ICIs had good efficacy in patients who underwent MIBC bladder preservation therapy.

We aimed to determine the predictive factors for the efficacy of bladder-sparing therapy combined with ICIs. PD-L1 expression is the most widely studied biomarker for immunotherapy. Studies on nivolumab or atezolizumab combined with radiotherapy have shown that PD-L1 expression may be a potential biomarker for ICIs with radiotherapy (20, 28). However, a study on durvalumab combined with radiotherapy for bladder-sparing therapy showed no correlation between baseline PD-L1 expression and clinical prognosis (21), which is similar to the findings of this study. Current bladder-sparing studies have shown that the relationship between PD-L1 and efficacy is controversial. The use of these agents should not be excluded in patients with UC that lack PD-L1 expression. Moreover, patients with stage T2 disease have always been the preferred population for TMT bladder-sparing treatment, so does in immunotherapy-based bladder-sparing treatment. Many studies have shown that the prognosis of bladder-sparing treatment for patients with T2 disease is better than that of treatment for patients with T3–T4 disease (29, 30).

In terms of safety, 35.7% of patients enrolled in this study experienced grade 3 or 4 AEs, and 8% experienced grade 3 or 4 IRAEs. Common AEs were fatigue, cystitis, nausea and vomiting, thrombocytopenia, and leukopenia. Hypothyroidism, skin pruritus, and fatigue were the main IRAEs. In bladder preservation studies on pembrolizumab combined with TMT, the incidence of grade 3–4 AEs was 21.4%–40% (14, 18), which was consistent with the findings of this study. However, the incidence of grade 3–4 AEs among patients undergoing ICI administration combined with radiotherapy was 13%–35% (15, 20, 21, 28), which suggested that combined bladder-preserving treatment without chemotherapy is more suitable for older adult patients who are frail and chemotherapy-ineligible. The AEs in the abovementioned study mainly included gastrointestinal toxicity, hematological toxicity, and endocrine toxicity, which were similar to those that occurred in this study.

This study has some limitations. First, it was a retrospective study performed at a single institution. Second, a relatively small number of patients was included. Future studies should include larger sample size, and randomized clinical studies should be conducted. Third, there is no unified standard for evaluating efficacy. In the existing research, there are many evaluation contents, including cCR, pCR, DFS, BIDFS, PFS, and OS, and a lack of long-term follow-up results. In this study, cCR was selected as the primary endpoint to evaluate short-term efficacy; however, long-term follow-up is needed to understand the long-term prognosis of patients.

## Conclusions

In this study, the addition of a PD-1 inhibitor improved the efficacy of traditional bladder-preservation therapy (TMT and TURBT plus radiotherapy) for patients with MIBC who are incompatible with RC. The treatment mode is feasible and safe, and chemotherapy and PD-L1 expression do not affect efficacy and prognosis. Despite controversies regarding this type of bladder-preservation therapy, it shows great potential.

## Data availability statement

The original contributions presented in the study are included in the article/**Supplementary Material**. Further inquiries can be directed to the corresponding author.

## Ethics statement

The study was conducted in accordance with the Declaration of Hensinki and approved by the appropriate ethics review board of the Second Xiangya Hospital of Central South University, Changsha, China. All methods were performed in accordance with the relevant guidelines and regulations, and informed consent was obtained from all subjects and/or their legal guardian(s).

## Author contributions

Conceptualization, JW, WZ, and YF; material support, YL; data acquisition, LZ, RX, YL, RZ; statistical analysis, JW; writing—draft, CX; revision, JW; editing, XL and YW; supervision, XL and YW. All authors commented on previous versions of the manuscript. All authors contributed to the article and approved the submitted version.
