# Supplementary material for: Real-world retrospective study of immune checkpoint inhibitors in combination with radiotherapy or chemoradiotherapy as a bladder-sparing treatment strategy for muscle-invasive bladder urothelial cancer
Source: Front Immunol. 2023 May 22;14:1162580. doi: 10.3389/fimmu.2023.1162580 (PMC10239884; doi:10.3389/fimmu.2023.1162580)
Supplement: Supplementary file 1 [file Table_1.docx]

Supplementary table 1: Multivariate analysis of clinical outcomes

| Variables | Multivariate analysis | |
| --- | --- | --- |
|  | HR | P value |
| **OS** |  |  |
| T stage | 3513.5 | 0.845 |
| PD-L1 Expression | 1.067 | 0.999 |
| ICI | 482.8 | 0.628 |
| Chemotherapy | 0.916 | 0.999 |
| **DFS** |  |  |
| T stage | 7.766 | 0.128 |
| PD-L1 Expression | 61796 | 0.987 |
| ICI | 4.558 | 0.290 |
| Chemotherapy | 0.000 | 0.983 |
